# Impact of ultrasound operator training on clinical pregnancy rates during embryo transfer: a retrospective cohort study

**DOI:** 10.3389/frph.2025.1743979

**Published:** 2026-02-13

**Authors:** Ranit Hizkiyahu, Michal Bezalel, Miri Godin, Chana Adler Lazarovits, Yaakov Bentov, Efrat Esh-Broder, Anat Hershko Klement

**Affiliations:** 1 The IVF Unit, Department of Obstetrics and Gynecology, Hadassah Mount Scopus-Hebrew University Medical Center, Jerusalem, Israel; 2 Faculty of Medicine, Hebrew University of Jerusalem, Jerusalem, Israel; 3 Department of Obstetrics and Gynecology, Faculty of Medicine, Hebrew University of Jerusalem, Jerusalem, Israel

**Keywords:** embryo transfer, *in vitro* fertilization, pregnancy, staff, hospital, ultrasound

## Abstract

**Introduction:**

While various aspects of the embryo transfer (ET) procedure have been studied for their potential impact on treatment outcomes, the influence of ultrasound (US) operator guidance during ET has not been extensively explored. Therefore, this study aims to investigate the impact of US guidance performed by well-trained versus untrained medical staff on the clinical pregnancy rate.

**Material and methods:**

This is a retrospective study that was conducted in a single university-affiliated IVF unit between February 2023 and April 2024. The study compared the clinical pregnancy rate between patients undergoing ET by an US operator versus an untrained operator.

**Results:**

A total of 951 embryo transfers were analyzed: 442 performed by trained operators (46.5%) and 509 by untrained operators (53.5%). Demographic characteristics were comparable. Main diagnosis, day of transfer, and mean number of embryos transferred were similar between groups (*p* = 0.2, 0.3, 0.4, respectively). The main outcome measure, clinical pregnancy crude rate, was similar (31.6% untrained vs. 31.4% trained, *p* = 0.9). Factors identified as associated with achieving a clinical pregnancy were maternal age (*p* < 0.01), endometrial thickness (*p* = 0.012), type (frozen vs. fresh) of embryos (*p* = 0.029), and embryonal age (*p* = 0.012). In a conditional logistic regression analysis, the US operator was not found to be a significant effector.

**Conclusions:**

The utilization of a trained versus untrained ultrasound operator during ET was not associated with a difference in clinical pregnancy rates.

## Introduction

Embryo transfer (ET) represents the final procedure in IVF treatment and plays a critical role in the entire process, thus significantly impacting the treatment outcome (1). Several factors before, during, and after embryo transfer have been described to have an influence on its success (2, 3). Some examples of positive contributory factors, which were reported to increase pregnancy rates, are using a soft catheter for insertion of the embryo, using a hyaluronic acid–containing transfer medium for the embryo, and using US-guided embryo transfer (3). The importance of using US guidance during embryo transfers was demonstrated in multiple studies and summarized in a review published by the Cochrane library (4). Transabdominal US guidance was compared with “clinical touch' during embryo transfer. The “traditional' method of embryo transfer—clinical touch—relied on the clinician's tactile senses to judge when the transfer catheter was in the correct position. The results showed that US-guided ET was associated with an increase in the chances of a clinical pregnancy (OR: 1.31, 95% CI: 1.17–1.45).

The role of the physician performing the procedure itself was investigated in a couple of previous publications. On the one hand, the extent of experience of the physician performing the ET (fellow vs. attending trained physician) was not proven to improve clinical outcomes. On the other hand, clinical pregnancy rates may vary depending on the individual transferring physician (5).

In 2017, the American Society for Reproductive Medicine published a guideline for embryo transfer that included the following recommendations: abdominal US guidance for embryo transfer, removal of cervical mucous, use of soft embryo transfer catheters, correct placement of the embryo transfer tip in the uterine cavity, and immediate ambulation once the embryo transfer procedure is completed (2). A recent overview of the technical aspects of US-guided ET, published by the European journal Human Reproduction Open, has added a recommendation of cleansing of the vagina/cervix prior to ET by using sterile water or saline and emphasized that the duration of the ET procedure affects success rates. A duration of a transfer of more than 120 s has shown a negative effect (6).

The US operator guiding the transfer has not been discussed in these guidelines, probably due to a paucity of well-designed studies assessing this factor. To the best of our knowledge, two studies have investigated this issue so far: the first, a retrospective review published in 2009 (7), and the second, a prospective randomized study published in 2014 (8). Neither showed a significant difference in the clinical outcome as stratified by the sonographer. In the current study, we aim to re-examine the impact of US guidance performed by a well-trained operator versus an untrained operator on achieving clinical pregnancy. Our hypothesis is that US operator training status does not influence clinical pregnancy rates. Our study adds value by including a larger sample size, representing a contemporary cohort, and adjusting for important confounders.

## Materials and methods

We conducted a retrospective study utilizing data sourced from a university-affiliated IVF clinic (Mount Scopus, MS). Patients included in the study underwent an embryo transfer between February 2023 and April 2024. The outcome was compared between patients who underwent the ET with US guidance performed by a trained operator (“exposed”) versus untrained medical staff (“unexposed”). A trained operator was defined as either one of the following: an US technician, a reproductive medicine senior physician, or a fellow in reproductive medicine. A non-trained operator was defined as either an operating room (OR) nurse or an out-patient staff nurse. These nurses do not have an US-specific training or education. The attendance of an US technician to assist in embryo transfers is based on staff availability as they are serving multiple roles in our institution. Whenever a physician or technician was not available to perform the US scan, the allocated OR nurse or the outpatient clinic nurse was asked to assist in performing the US for the procedure. Because technician availability was determined by unrelated institutional duties, this process functioned as a “natural experiment,” whereby some embryo transfers were performed with US guidance by a trained technician and others by a nurse assigned on an availability basis. In our IVF unit, only trained reproductive medicine physician or senior reproductive medicine fellows perform ETs. Individual variations between physicians are assessed and monitored every 6 months and they show no significant interphysician difference. Uterine factor infertility cases, embryo transfers from surgically retrieved sperm, and procedures with incomplete staff records were excluded from the analysis. In the non-donor cycles that were included, the transfer was performed within the same year of oocyte retrieval.

A computerized database including the following variables was established: trained versus untrained operator, age of patient, smoking status, BMI, donor/self-gametes and indication for treatment, frozen/fresh embryo transfer, embryo age at transfer, and number of embryos transferred. The indication for treatment was categorized into three groups: male factor, donor gametes, and non-male factor (including diagnoses of PCOS/anovulation, unexplained infertility, advanced maternal age, mechanical factor, diminished ovarian reserve, and PGT).

The primary outcome measure was clinical pregnancy.

### Endometrial preparation protocols

The protocols for endometrial preparation were determined by the treating physician. In the natural cycle group, the participants were monitored for ovulation using serial US scans and blood tests for LH, estradiol, and progesterone serum concentrations, starting on day 2–3 of the cycle. In these ovulatory cycles, embryo transfer was scheduled according to the LH surge and the embryo's age, as described in detail in a previous publication (9). In the modified natural cycles, hCG was used to induce ovulation once the dominant follicle reached >15 mm and the endometrial lining was >7 mm. Luteal phase support was initiated on the day of ovulation, using oral dydrogesterone (Duphaston. Abbott healthcare products b.v., Netherlands) of 10 mg, two to three times a day, or vaginal progesterone suppositories (Utrogestan. Besins-international laboratories, France) of 400 mg, two to three times a day. Luteal phase support was continued until 10 weeks of gestation. In the medicated frozen embryo cycles, oral estradiol valerate (Estrofem. Novo Nordisk a/s, Denmark) was given at a daily dose of 6 mg, starting on day 1–3 of the cycle. When the endometrial thickness reached >7 mm width, vaginal progesterone suppositories (400 mg, three times a day) were added and the transfer scheduled to +1 days of progesterone exposure. Progesterone levels were monitored on the day before or on the same day of the transfer. If the progesterone level was lower than 10 ng/mL, intramuscular (IM) progesterone injections of 50 mg (Prontogest. IBSA Farmaceutici, Italy) were added, with a frequency every third day (10, 11). Treatment continued until 10 weeks of gestation. The follow-up of all participants was continued in the IVF unit until 6–7 weeks of pregnancy. Clinical pregnancy was defined as an intrauterine gestational sac documented by transvaginal US. Following 6–7 weeks of pregnancy, the patients were referred to general community-based ObGyn clinics.

### Statistical methods

#### Data analysis

All analyses were performed using the IBM SPSS statistics for Windows (version 24.0, IBM Corp., Armonk, NY, USA). As the data were normally distributed, we used the Student's *t*-test for comparisons involving continuous variables. A chi-square test or Fisher's exact test was used for the comparison of rates and proportions. BMI was categorized into ≤30 and >30. A logistic regression analysis was performed for modeling clinical pregnancy: factors that demonstrated a statistically significant correlation with clinical pregnancy were integrated. All *P*-values were two-tailed and considered significant at less than 0.05.

### Data availability

Data regarding current research will be available upon request.

## Results

A total of 951 embryo transfers were included in the study; of these, 442 transfers were performed with trained US guidance (46.5%): of these 442, 380 (40%) were technician guided and 62 (6.5%) were physician guided. The remaining 509 (53.5%) transfers were performed with untrained US guidance. A total of 22 out of the 951 transfers were documented as difficult to perform; of these, 12 were guided by an untrained operator and 10 were guided by a trained operator.

Patient characteristics in each group are described in Table 1. Most of these characteristics were comparable between the groups. Maternal age reflected a relatively young population (33.8 ± 6.6 in the trained group, 33.8 ± 6.6 in the untrained group, *p* = 0.942). The groups presented a statistically different distribution of the type of embryo transferred (*p* < 0.01)—frozen vs. fresh—and endometrial thickness (9.35 ± 1.8 mm vs. 9.74 ± 2.2 mm, *p* < 0.01).

**Table 1:** Group characteristics according to the type of US guidance (trained versus untrained).

Group characteristics		Trained (*n* = 442, 46.5%)	Untrained (*n* = 509, 53.5%)	*p*
Maternal age (mean, years)		33.8 ± 6.6	33.8 ± 6.6	0.942
BMI (mean, kg/m^2^) categorized^b^	>30	79 (21.6%)	98 (22.8%)	0.732
	≤30	286 (78.4%)	331 (77.2%)	
Endometrial thickness (mean, mm)		9.35 ± 1.8	9.74 ± 2.2	*p* < 0.01^a^
Parity	Multiparous	86 (19.5%)	114 (22.4%)	0.267
	Nulliparous	356 (80.5%)	395 (77.6%)	
Type of embryo transferred	Frozen	368 (83.6%)	344 (68.1%)	*p* < 0.01^a^
	Fresh	72 (16.4%)	161 (31.9%)	
Number of embryos transferred	1	331 (75.2%)	365 (72.3%)	0.440
	2	108 (24.5%)	137 (27.1%)	
	3	1 (0.2%)	3 (0.6%)	
Embryonal age	Cleavage	186 (42.1%)	216 (42.6%)	0.350
	Morula (day 4)	83 (18.8%)	78 (15.4%)	
	Blastocyst (day 5–6)	173 (39.1%)	213 (42%)	
Diagnosis	Male factor	146 (33.7%)	157 (31.3%)	0.221
	Donor	28 (6.5%)	22 (4.4%)	
	Non-male	322 (59.8%)	259 (64.3%)	

a A statistically significant difference.

b Data based on 794 cases.

We conducted a univariate analysis to explore the association between achieving a clinical pregnancy and possible confounders (Table 2). As for the main outcome measure, there were 300 clinical pregnancies, 139 (31.4%) in the trained group and 161 (31.6%) in the untrained group, *p* = 0.952 (ratio 0.994, 95% CI: 0.82–1.2). Pregnancy rates were similar when they were further subdivided into physician versus technician guidance (*p* = 0.905). The factors identified as associated with achieving a clinical pregnancy were maternal age, endometrial thickness, type of embryo (frozen vs. fresh), and embryonal age (Table 2).

**Table 2:** Univariate analysis for clinical pregnancy distribution according to the type of US guidance and other related variables.

Variables		Clinical pregnancy achieved	Not pregnant	*p*
US guidance training	Trained	139 (31.4%)	303 (68.6%)	0.952
	Untrained	161 (31.6%)	348 (68.4%)	
Maternal age (mean, years)		33.0 ± 6.5	34.2 ± 6.6	*p* < 0.01^a^
BMI (mean, kg/m^2^) categorized	>30	47/254 (18.5%)	130/540 (24.1%)	0.08
Endometrial thickness (mean, mm)		9.81 ± 1.9	9.44 ± 2.1	0.012^a^
Parity (previous deliveries)	Multiparous	62 (31%)	138 (69%)	0.852
	Nulliparous	238 (31.7%)	513 (68.3%)	
Type of embryos returned	Frozen	238 (33.4%)	474 (66.6%)	0.029^a^
	Fresh	60 (25.8%)	173 (74.2%)	
Number of embryos transferred	1	214 (30.7%)	482 (69.3%)	0.695
	2	83 (33.9%)	162 (66.1%)	
	3	1 (25%)	3 (75%)	
Embryonal age	Cleavage (day 1–3)	113 (28.1%)	289 (71.9%)	0.012^a^
	Morula (day 4)	44 (27.3%)	117 (72.7%)	
	Blast (day 5–6)	143 (37%)	243 (63%)	
Diagnosis	Male factor	107 (35.3%)	196 (64.7%)	0.181
	Donor	18 (36%)	32 (64%)	
	Non-male factor	172 (29.6%)	409 (70.4%)	

a A statistically significant difference.

In a stepwise (conditional) logistic regression model (Table 3) integrating our variable of interest (US-guidance type) as an obligatory variable along with maternal age, endometrial thickness, type of embryo (frozen vs. fresh), and embryonic age, we found that maternal age, endometrial thickness, and embryonic age were all significant predictors of clinical pregnancy. A thicker endometrium was a positive predictor of clinical pregnancy (*p* = 0.016), while advanced maternal age and non-blastocyst embryos were negatively associated with the chance of achieving pregnancy (0.045 and 0.03, respectively). US operator was not found to be a significant effector.

**Table 3:** Conditional logistic regression model for clinical pregnancy.

Parameter	B	S.E.	Sig	Adjusted OR	95% CI for OR	
					Lower	Upper
Endometrial thickness (mm)	0.082	0.034	0.016*	1.085	1.015	1.160
Maternal age	−0.022	0.011	0.045*	0.978	0.957	0.999
Embryonic age	0.184	0.543	0.03*	1.202	1.029	1.405
Trained guidance	0.014	0.144	0.92	1.015	0.766	1.345

* Significance level ≤0.05.

## Discussion

In this retrospective study, we aimed to investigate whether the utilization of a trained US operator during embryo transfer impacts clinical pregnancy rates. The study revealed that clinical pregnancy rates were comparable irrespective of whether the US operator was trained or untrained. As expected, we found that younger maternal age, thicker endometrial lining, and transfer of blastocyst stage embryos were all associated with higher clinical pregnancy rates.

While there is a substantial body of literature emphasizing the importance of US guidance during embryo transfer and other technical aspects of the procedure, we identified only two studies that assessed the impact of trained vs untrained US operator on treatment outcome. In 2009, Harris and his colleagues were the first to raise the question whether the clinical experience and level of US training correlate with IVF outcomes. They conducted a retrospective study of 319 women who underwent embryo transfer: 201 (63%) underwent ET, with the reproductive endocrinology and infertility (REI) fellow performing the US, and 118 (37%) underwent the procedure with the help of a medical assistant. Clinical pregnancy rates and live birth rates were similar between groups (*p* = 0.82 and 0.99, respectively) (7). However, their study had several limitations, including the small number of patients, treatment at different time periods, lack of information regarding embryo age, and other potential confounding factors.

More recently, a prospective randomized study conducted by Rinaldi et al. compared the results of embryo transfer performed by an experienced US guidance (a physician) and non-experienced guidance (an untrained midwife). The study included a total of 553 patients and found no differences between groups with regard to the technique itself—unsatisfactory visualization and difficulty in transfer—and with regard to treatment outcomes—pregnancy rate and extrauterine pregnancy rate (8).

In accordance with the aforementioned studies, our findings further confirm that the presence of a trained US operator during embryo transfer does not significantly impact clinical pregnancy rates. Several factors may explain this observation. First, the procedure is team-based. The physician performing the catheter insertion is the ultimate decision-maker, using the US image as a guide, and may compensate for suboptimal imaging. Furthermore, in cases where an untrained operator encounters difficulty achieving optimal visualization, the physician performing the procedure may intervene by taking over the US probe or providing real-time guidance. This collaborative approach could mitigate any potential negative effects of operator inexperience. In addition, the fundamental technical skill required for basic transabdominal guidance to visualize the catheter tip may have a low threshold for competence, easily met by nursing staff with a brief instruction.

Second, it is possible that the exact placement location of the embryo within the uterine cavity may not be as critical to treatment success as previously assumed. Embryo migration in the uterine cavity following embryo transfer was already illustrated in a couple of previous publications (12–14). This could allow for a degree of flexibility in executing the procedure, even under suboptimal visualization conditions.

The strength of our study lies in being the largest to date to investigate a routinely encountered clinical question that has been previously underexplored. The availability of the US operator was a random event and was not controlled by the performing physician. This operator availability serves as a natural experiment, reducing allocation bias. Our study has several limitations. First, its retrospective design inherently limits the ability to control for all potential confounders. Specifically, duration of infertility and the number of previous IVF attempts were not included in the analysis. We reported clinical pregnancy rates, without reporting live birth outcome data. Embryo quality data were unavailable and may represent residual confounding. In addition, the study defines “trained” operators as technicians, senior physicians, or fellows, but heterogeneity within the trained operator group could have influenced outcomes. Another consideration is the possibility that specific US operators were preferentially assigned to manage difficult cases, potentially introducing selection bias, as these patients might have had a reduced likelihood of achieving conception. Nevertheless, only 22 out of 951 transfers were categorized as difficult (according to the operator's impression), with these cases being nearly evenly distributed between the study groups. Therefore, it is unlikely that difficult transfers significantly biased the results.

Finally, our study was conducted in a single-center setting. On the one hand, standardized protocols are a strength, but on the other hand, it could limit generalizability.

## Conclusion

In this large retrospective study, the training status of the US operator was not associated with clinical pregnancy rates after embryo transfer. In settings with routinely available trained sonographers/physicians, this remains best practice. However, in resource-constrained settings or when staffing is limited, using an untrained operator to hold the probe does not appear to compromise outcomes, based on the evidence gathered in this study and prior evidence. Our results may allow for more flexible staffing models and increased availability and accessibility of procedure personnel.

## Data Availability

The raw data supporting the conclusions of this article will be made available by the authors without undue reservation.
